# Multidimensional assessment of anxiety through the State-Trait Inventory for Cognitive and Somatic Anxiety (STICSA): From dimensionality to response prediction across emotional contexts

**DOI:** 10.1371/journal.pone.0262960

**Published:** 2022-01-25

**Authors:** Filipa Barros, Cláudia Figueiredo, Susana Brás, João M. Carvalho, Sandra C. Soares

**Affiliations:** 1 William James Center for Research (WJCR), Department of Education and Psychology, University of Aveiro, Aveiro, Portugal; 2 Center for Health Technology and Services Research (CINTESIS), Department of Education and Psychology, University of Aveiro, Aveiro, Portugal; 3 Centre for Mechanical Technology and Automation (TEMA), University of Aveiro, Aveiro, Portugal; 4 Research Unit on Governance, Competitiveness and Public Policies (GOVCOPP), University of Aveiro, Aveiro, Portugal; 5 Department of Electronics, Telecommunication and Informatics (DETI), University of Aveiro, Aveiro, Portugal; 6 Institute of Electronics and Informatics Engineering (IEETA), University of Aveiro, Aveiro, Portugal; 7 Department of Clinical Neuroscience, Division of Psychology, Karolinska Institute, Stockholm, Sweden; European University of Rome, ITALY

## Abstract

The assessment of mal-adaptive anxiety is crucial, considering the associated personal, economic, and societal burden. The State-Trait Inventory for Cognitive and Somatic Anxiety (STICSA) is a self-report instrument developed to provide multidimensional anxiety assessment in four dimensions: trait-cognitive, trait-somatic, state-cognitive and state-somatic. This research aimed to extend STICSA’s psychometric studies through the assessment of its dimensionality, reliability, measurement invariance and nomological validity in the Portuguese population. Additionally, the predictive validity of STICSA-Trait was also evaluated, through the analysis of the relationship between self-reported trait anxiety and both the subjective and the psychophysiological response across distinct emotional situations. Similarly to previous studies, results supported both a four-factor and two separated bi-factor structures. Measurement invariance across sex groups was also supported, and good nomological validity was observed. Moreover, STICSA trait-cognitive dimension was associated with differences in self-reported arousal between groups of high/low anxiety, whereas STICSA trait-somatic dimension was related to differences in both the subjective and psychophysiological response. Together, these results support STICSA as a useful instrument for a broader anxiety assessment, crucial for an informed diagnosis and practice.

## Introduction

Anxiety is an emotional response following the anticipation of a threatening stimulus, either real or perceived [1]. It encompasses a complex and multidimensional phenomenon associated with muscular tension, subjective distress, enhanced vigilance concerning future threat and avoidance behaviors, which prepare the body for action and adaptive behavior [1, 2]. Nevertheless, anxiety is often associated with great distress and poor health outcomes when persistent and/or significantly intense [1, 3]. In fact, anxiety disorders are one of the most observed groups of psychopathologies, with an estimated prevalence of 7.3% globally (4.8–10.9%; see [4]), and are often associated with other mental disorders [5], and health problems, such as cardiovascular diseases [6]. Furthermore, anxiety symptomatology is characterized by great inter and intra-individual heterogeneity, along with variable symptom trajectories over time [7]. Altogether, these factors complexify the assessment, diagnosis and treatment of non-adaptive anxiety, catalyzing the associated societal, personal and health care costs and burden [3, 8].

Self-report instruments have been extensively used as a simple, brief and non-invasive method of assessing anxiety across contexts [9]. Although many of them present satisfactory psychometric properties [10, 11], most present several limitations regarding, for instance, the ability to discriminate anxiety from depression [12], and the ability to embrace multiple anxiety dimensions. To surpass some of these difficulties and to provide a multidimensional assessment of anxiety, Ree and colleagues [13] developed the *State-Trait Inventory for Cognitive and Somatic Anxiety* (STICSA). This instrument encompasses 42 items divided in two forms of 21-items each: the state anxiety (STICSA-State) and the trait anxiety (STICSA-Trait) form. State anxiety is conceptualized as the anxiety response experienced at the moment, i.e., in a limited period; in turn, trait anxiety corresponds to individual differences regarding anxiety as an emotional response relatively stable in time [11, 14]. STICSA also addresses the cognitive and somatic components of anxiety within each one of its forms; the cognitive dimension encompasses symptoms such as worry, intrusive thoughts and rumination (e.g., “Feel agonized over problems”), while the somatic component refers to psychophysiological activation, including palpitations or excessive sweating (e.g., “Heart beats fast”). Discerning between these four dimensions can be critical to characterize anxiety profiles and to predict people’s response in certain contexts. In fact, people can present distinct cognitive and somatic symptomatology [15].

STICSA has been supported as an adequate measure of anxiety across healthy and clinical samples, demonstrating good nomological validity [13, 16–18]. Furthermore, most psychometric studies of STICSA support the construct validity of the two-factor structure within STICSA-Trait and STICSA-State forms [13, 17–21], as well as the four-dimension structure [16, 17, 22]. Yet, past studies still evidence mixed findings and have left open questions regarding STICSA’s dimensionality (see [20]). Therefore, it is crucial to extend STICSA’s psychometric studies to other settings and cultures, as well as to confirm its adequacy and utility across contexts. Also, although STICSA provides important information about self-reported anxiety symptomatology, the relationship between the reported symptoms and other dimensions of the emotional response of the individuals in certain contexts remains poorly understood. The accordance between the multiple components of the emotional response (e.g., subjective and psychophysiological responses) is often low [23], with each component providing unique and complementary information [24]. Nevertheless, in the absence of more objective measures and considering the frequent time and financial constraints observed across contexts, self-report is often one of the few and more reliable tools to assess anxiety. Therefore, studying how scores in the four dimensions of STICSA are able to predict individuals’ emotional response beyond the subjective domain is crucial to obtain more accurate information about people’s anxiety profile, health and well-being.

Considering this mindset, we propose two main aims for our research. First, we sought to extend the validation studies of STICSA, while adapting this instrument for the Portuguese population. Second, we aim to assess the relationship between STICSA-Trait scores and the individuals’ subjective and psychophysiological responses to emotional stimulation. Since trait anxiety reflects relatively stable differences between people in the proneness to experience symptoms of anxiety [14], it may be especially useful to predict an individual’s emotional response across contexts. With these aims, we intend not only to support the construct validity of STICSA, but also to maximize and integrate information about different dimensions of anxiety across emotional contexts. Moreover, this research seeks to provide a better comprehension of how distinct dimensions of self-reported trait anxiety predicts an individual’s emotional response in different emotional contexts, including how these scores relate to a biomarker of autonomic dysfunction and psychopathology, namely Heart Rate Variability (HRV). This can be very critical knowledge, not only considering research settings but also clinical practice, given that self-report is often the most accessible and cost-effective tool to support assessment, diagnosis and monitoring of anxiety.

## Study 1: Psychometric study of STICSA

Previous psychometric studies of STICSA assessed and supported factorial validity of the state-trait and cognitive-somatic distinction by computing full scale confirmatory factorial models and observing, for instance, a four-factor correlated model [16, 17, 22], or a hierarchical model with a global anxiety factor supported in the four factors (state-cognitive, state-somatic, trait-cognitive and trait-somatic anxiety; [17]). Dimensionality was also established by estimating separate models, namely a two-factor model for state [13, 17–20] and trait forms [13, 17–21]. Furthermore, studies of measurement invariance across gender and age groups [18, 19], as well as a multi-method approach with an informant within friendship dyads of trait anxiety [21], gave further support for the quality of the STICSA measurement. Previous studies have also revealed good convergent and nomological validity [13, 16–19, 22]. Nevertheless, STICSA evidenced medium to high correlations with depression measures (r = .42 - .67), especially when considering the correlation with cognitive anxiety (r > .48; [16, 18, 22]), despite of being better at discriminating anxiety from depression in comparison with, for instance, the State-Trait Anxiety Inventory (STAI; [11]), an instrument broadly used to measure anxiety in its state and trait dimensions (e.g., [16]). STICSA has been proven to be, therefore, a suitable instrument to measure anxiety in general and clinical populations, but it is necessary to extend its psychometric studies to better characterize the instrument’s factor structure, validity, and its adequacy across cultures and settings. In order to assess STICSA’s psychometric properties in the Portuguese population, its dimensionality, reliability, measurement invariance and nomological validity were assessed in Study 1.

### Materials and methods

#### Participants

The inclusion criteria included: 1) Age ≥18 years old; 2) Portuguese nationality; 3) Currently residing in Portugal. A final sample of 1153 Portuguese adults (753 females; 65.3%) from different regions of Portugal was collected. Age ranged from 18 to 78 years old (M = 29.47; SD = 13.70). Most of the participants were higher education students (n = 658; 57.2%) or active employed individuals (n = 338; 29.4%). Most of the participants were single (n = 825; 71.7%) and did not have children (n = 872; 75.7%). Furthermore, 13.4% of the participants (n = 154) reported having a psychological/psychiatric problem, and 6.2% (n = 71) reported being monitored/receiving treatment regarding those problems. For further information about sample’s characteristics please refer to Table 1. All participants were informed about the voluntary nature of their participation and the anonymity of collected data. The study was approved by the Ethics and Deontology Committee of the University of Aveiro (ref. 08/2018) and was performed according to the guidelines of the Declaration of Helsinki and the American Psychological Association.

**Table 1:** Sample’s demographic information.

		Total sample (n = 1153)		Women (n = 753)		Men (n = 400)	
Age		M = 29.47	SD = 13.70	M = 29.65	SD = 13.39	M = 29.15	SD = 14.28
		N	%	N	%	N	%
**Sex**	Female	753	65.3	753	100.0	-	-
	Male	400	34.7	-	-	400	100.0
**Education**	Basic education	44	3.8	30	4.0	14	3.5
	Secondary education	555	48.2	331	44.0	224	56.0
	Higher education	536	46.5	387	51.5	149	37.3
	Other	17	1.5	4	0.5	13	3.3
**Occupation**	Students	658	57.2	412	54.8	246	61.7
	Active employment	338	29.4	235	31.2	103	25.9
	Both student and employee	47	4.1	29	3.9	18	4.5
	Domestic	6	0.5	6	0.8	-	-
	Retired	38	3.3	21	2.8	17	4.3
	Unemployed	29	2.5	24	3.2	5	1.3
	Other	35	3.0	25	3.3	10	2.5
**Marital status**	Single	825	71.7	527	70.1	298	74.7
	Married	235	20.4	158	21.0	77	19.3
	Cohabiting	47	4.1	35	4.7	12	3.0
	Divorced	37	3.2	26	3.5	11	2.8
	Widower	7	0.6	6	0.8	1	0.3
**Have children?**	Yes	280	24.3	192	25.5	88	22.0
	No	872	75.7	560	74.5	312	78.0
**Psychiatric problems**	Yes	154	13.4	120	16.0	34	8.5
	No	997	86.6	631	84.0	366	91.5
**Currently monitored/ receiving treatment for psychiatric problems**	Yes	71	6.2	55	7.3	16	4.0
	No	1075	93.8	694	92.7	381	96.0

*Note*. Considering the measurement invariance analyses, descriptive data are also presented for groups divided by sex (women/men). Whenever the total value of cases does not correspond to 1153, it is due to missing values. Only valid answers are presented in this table.

#### Instruments

In a first step, the 42 items of STICSA were translated to the Portuguese language by the research team, followed by a retroversion conducted by an English native speaker. The two versions were then discussed to obtain a preliminary version of the scale. This version was tested in a group of individuals to assess clarity and to identify possible misinterpretations associated with the items wording and instructions, as well as to evaluate the average response time of the overall protocol. After collecting their feedback, the research team made minor adjustments, finalizing the process of translation and adaptation of the 42 items (21 items per subscale).

Considering the procedure followed by one of the original psychometric studies of STICSA [16], STAI [11], the Depression, Anxiety and Stress Scales–Depression scale (DASS-D; [25]) and the Positive and Negative Affect Schedule (PANAS; [26]) were selected to evaluate nomological validity. These instruments have been widely used to measure anxiety, depressive symptomatology, as well as positive and negative affect, respectively, aspects that were on the basis of STICSA’s development. In fact, STICSA was developed considering a state/trait distinction similar to STAI, and it sought to surpass one of STAI’s greatest limitations, related to its high associations with depression and low positive affect [27, 28]. The protocol was then firstly constituted by a sociodemographic questionnaire, with relevant information for the sample’s characterization, followed by STICSA (42 items), STAI (40 items), PANAS (20 items) and DASS-D (7 items).

*State-Trait Inventory for Cognitive and Somatic Anxiety (STICSA)*. The STICSA [13] is a self-report instrument that measures anxiety considering four dimensions: state, trait, cognitive and somatic anxiety. It is divided in two forms, each one with the same 21 items: one evaluating anxiety in its state dimension (“how do you feel right now”; STICSA-State) and the other evaluating its trait dimension (“how often this is true for you”; STICSA-Trait). Both forms evaluate cognitive and somatic components of anxiety, with items being rated in a 4-point scale that ranges from 1 (Not at all) to 4 (Very much; see [16]).

*State-Trait Anxiety Inventory (STAI—Form Y)*. The STAI—Form Y [11] is a psychological assessment instrument that measures anxiety. This scale encompasses two self-report scales of 20 items each: the state anxiety scale (STAI-Y1), which assesses how the individual feels at the moment; and the trait anxiety scale (STAI-Y2), assessing how the individual generally feels. Participants are asked to respond to each item using a four-point scale that ranges from 1 (Nothing at all) to 4 (Very much so) in the state scale, and from 1 (Almost never) to 4 (Almost always) in the trait form. This instrument was adapted and validated for the Portuguese population by Santos and Silva [29] and further psychometric studies supported its adequacy as a measure of anxiety [30].

*Positive and Negative Affect Schedule (PANAS)*. The PANAS [26] is a self-report instrument that measures positive and negative affect. This scale encompasses 10 items evaluating positive affect and 10 items evaluating negative affect. Each item corresponds to an emotion and the individual is asked to indicate how much he/she felt that emotion in the last weeks, using a scale ranging from 1 (Very slightly or not at all) to 5 (Extremely). PANAS was adapted for the Portuguese population by Galinha and Pais-Ribeiro [31] confirming the original factor structure of the instrument, and its adequate psychometric properties.

*Depression*, *Anxiety and Stress Scales–Depression scale (DASS-D)*. The DASS-21 is a self-report instrument comprising three scales evaluating anxiety, depression and stress, with 21 items distributed equally in each dimension [25]. Response scale encompasses a four-point scale that ranges from 0 (Did not apply to me at all) to 3 (Applied to me very much or most of the time), considering the frequency or severity of the negative emotional symptoms experienced in the last week [25]. This instrument was adapted for the Portuguese population by Pais-Ribeiro and colleagues [32], revealing adequate psychometric properties for the three subscales in an adult sample. In the current study, only the depression subscale was used.

#### Procedure

The participants’ recruitment and data collection followed a mixed-mode survey procedure [33], namely: 1) data collection in paper, mostly carried out in classroom context (targeting higher education students) and with older people of the general population; and 2) data collection through a digital platform (targeting the general population). In both modes, participants were informed about the study mainly through e-mail, class visits, flyer distribution, and social networks. All participants received detailed information about the study and gave their informed consent before their participation (by consenting and proceeding with the protocol in the digital platform, or by providing written informed consent when data collection was performed in paper). There was no compensation for their participation in the study. The protocol took, on average, between 15 and 20 minutes to complete.

#### Analytic procedures

The Mplus 8 version [34] was used to conduct Confirmatory Factorial Analysis (CFA), both in one group and multi-group (measurement invariance). The IBM SPSS Statistics (version 23) was employed for descriptive and reliability analyses. Additionally, R package’s Amelia II [35] was used to handle missing data. The missing data analysis was performed considering a sample higher than 1000, through a multiple imputation procedure. The used algorithm first created a bootstrapped version of the original data, through which estimated the sufficient statistics by Expected Maximization (EM) on a bootstrapped sample, and then imputed the missing values of the original data using the estimated sufficient statistics. This procedure was carried out by producing five datasets that were aggregated afterwards, assuming data as ordinal; in fact, three of the four measurement instruments with imputed missing data had 4-point answer scales, and the remaining instrument had a 5-point scale that ought to be considered ordinal as well. The imputation procedures were performed separately for each instrument. All participants presenting more than 10% of missing values in an instrument were excluded from the final sample [36]. The maximum imputed by variable was 1.2%, and the overall imputation encompassed less than 0.2% of total responses in the dataset.

The CFA were computed using a categorical robust Weighted Least Square Estimator (WLSMV) developed by Muthén and Muthén [34]. This estimator has been suggested as the one that best performs when testing categorical data [37, 38]. The assessment of model fit adjustment was based on robust chi-square statistic, as well as in the following fit indexes: Comparative Fit Index (CFI), Tucker and Lewis Index (TLI), Root Mean Square Error of Approximation (RMSEA), and Standardized Root Mean Residual (SRMR). Given the sample size of the present study, the interpretation of chi-square statistic was less helpful, since a non-significant result would not be expected [39]. Therefore, the model assessment assumed the following cut-off points: 1) CFI and TLI values higher than .95; and 2) RMSEA and SRMR values equal or less than .07 [38, 40].

A relevant dimension in psychometric validation studies is the equivalence of the measurement scale across different groups, i.e., to provide evidence that the same items are composing the same constructs in different groups of individuals, a condition that supports the accuracy of the group statistics [38, 41]. In this line, several studies suggest that anxiety disorders are more prevalent in women than in men (e.g., [42]). Therefore, sex invariance was tested in our sample, considering both state and trait models separately. Measurement invariance was assessed by testing several constrained models. First, it was assessed for each group separately, following three increasingly constrained models to compare the two groups regarding configural, metric, and scalar invariance [38, 41]. Model fit decision was assessed considering the parameters previously mentioned, and the comparison between the constrained models was done considering the difference in robust chi-square (chi-square difftest), being this option the most recommended when using the WLSMV estimator [43].

Reliability analysis was supported in McDonald’s Omega, which accounts for the effective results from the CFA analysis conducted [44, 45]. Cronbach alpha was also computed, as it is the most common internal consistency coefficient with Likert-type response scales, allowing the comparison between different studies [46]. Lastly, convergent and concurrent validity were observed through Pearson correlation [47]. The interpretation of the correlation was supported considering Cohen´s suggestion: small effect [.10- .30[, medium effect [.30 - .50[, and large effect: [.50–1] [48].

### Results

#### Dimensionality analysis

According to the procedures used to develop STICSA, as well as to the constructs it is meant to reflect, the dimensionality analysis can approach the full model or divide the analysis by trait and state forms. The full models are expected to express an extensive degree of multicollinearity since the items of state and trait have the same wording. Nevertheless, the dimensionality analysis assumed and explored both options. The CFA of the full model of STICSA scale (42 items) was assessed through five concurrent models:

- (MF1) a unidimensional model with all items loading in one factor;
- (MF2) a four correlated factor model with the state-cognitive, state-somatic, trait-cognitive and trait-somatic dimensions;
- (MF3) a four-factor model including state-cognitive, state-somatic, trait-cognitive and trait-somatic factors, as well as a second-order anxiety factor;
- (MF4) a four-factor model including state-cognitive, state-somatic, trait-cognitive and trait-somatic factors, as well as second-order state and trait factors;
- (MF5) a four-factor model including state-cognitive, state-somatic, trait-cognitive and trait-somatic factors, as well as a second-order state and trait factors and a third-order anxiety factor.

The MF4 and MF5 models were tested in the present study since they reflect the most complex models underlying the instrument’s structure, considering the somatic and cognitive dimensions within higher-order trait and state dimensions (MF4), as well as a third higher-order anxiety factor (MF5). Considering that all items of state and trait forms are exactly the same, with differences only in the scales’ instructions [16], the error terms were allowed to correlate between the mirror items.

All five models showed acceptable to good fit indexes. The decision about model adequacy was based on the results of the selected fit indexes (CFI, TLI, RMSEA and SRMR), since the results for the chi-square test were statistically significant, as expected due to the sample size [39]. Results showed that the four-factor model with second-order state and trait factors (MF4), as well as the three-order factor model (MF5) presented good adjustment. The model with four-correlated factors (MF2) was the one with the best overall adjustment within the full models (Table 2).

**Table 2:** Goodness of fit statistics for all concurrent models (n = 1153).

Models	χ^2^	df	CFI	TLI	SRMR	RMSEA (95% CI)
**Full models** ^a^						
MF1—One factor	5878.459*	798	.916	.909	.089	.074* (.073 - .076)
MF2- Four correlated factors	**2448.328** *	**792**	**.973**	**.970**	**.050**	.045* **(.043 - .045)**
MF3- Four correlated factors and one second order anxiety factor ^b^	6427.093*	795	.907	.899	.097	.078* (.077-.080)
MF4—Four factors and two 2^nd^ order correlated factors (State-Trait)^b^	3622.462*	794	.953	.949	.067	.056* (.054 - .058)
MF5—Three order factors (4 first order-2 second order—1 third order) ^b^	3643.984*	793	.953	.949	.067	.054* (.054–0.58)
**State models**						
MS1—One factor	1850.142*	189	.877	.863	.084	.087* (.084 - .091)
MS2—Two factors	**817.260** *	**188**	**.953**	**.948**	**.051**	**.054 (.050 - .058)**
MS3—One second order factor	4410.885*	189	.687	.652	.141	.139* (.136 - .143)
**Trait models**						
MT1—One factor	2769.272*	189	.889	.877	.082	.109* (.105 - .112)
MT2—Two factors	**1032.127** *	**188**	**.964**	**.960**	**.044**	.062* **(.059 - .066)**
MT3—One second order factor	6979.225*	189	.709	.677	.146	.177* (.173 - .180)

*Note*. Statistics: Chi-square (χ^2^); Comparative Fit Index (CFI); Tucker and Lewis Index (TLI); Standardized Root Mean Squared Residual (SRMR); Root Mean Square Error of Approximation (RMSEA).

^a^ all full models have correlated error terms between similar state and trait items

^b^ models with latent variable covariance matrix not positive definite due to linear dependency between two or more latent variables.

* p < .001.

The three models with good fit indexes were theoretically sound, with these results supporting the factorial structure proposed in the development of STICSA. However, these models presented very high correlations between the symmetric latent variables in state and trait forms. For instance, the MF2 model showed a correlation of .817 between the two dimensions of somatic anxiety and .916 between the two dimensions of cognitive anxiety. In general, the trait and state dimensions were highly associated in all models, suggesting that the respondents did not completely separate these two forms of anxiety.

Considering these findings, as well as the complexity of the models when all the mirror items were allowed to have their error terms correlated, separated models for STICSA-State and STICSA-Trait were tested. This option enables further assessment of the hypothesized models and their validity to be conducted in simpler models, respecting the assumption of local independence [49], as well as assuring the parsimony of the models by, for instance, not increasing the parameters to be estimated [39]. Different concurrent models were compared for each form. Three models were computed both for state and trait forms: (MS1 and MT1) a unidimensional model with all items loading in one factor; (MS2 and MT2) a two-factor model including somatic and cognitive factors within state or trait anxiety; and (MS3 and MT3) a two-factor model for somatic and cognitive dimensions explaining a second-order factor for state or trait anxiety. Once more, the MS3 and MT3 models were not tested in previous studies but were tested in the present study since they reflect a more complex model of the instrument’s structure. Results of the CFA performed for both trait and state models showed that the best overall adjustment was observed for the models with two factors (somatic and cognitive anxiety; MS2 and MT2). The results from the selected fit indexes for the unidimensional and the second-order factor model were below .95 for CFI and TLI and above .07 for SRMR and RMESEA, being these the suggested cut-off points for the models to be considered acceptable (Table 2).

The factor loadings for all items in both state and trait models were significant (p < .001). Standardized loadings for the state anxiety model ranged from .486 to .787 regarding the somatic factor and from .404 to .821 in the cognitive factor. Accordingly, on average, a square multiple correlation of .443 was observed for the somatic factor (.236≤SMC≤.620), and of .516 for the cognitive factor (.163≤SMC≤.675). Standardized loadings for the trait models ranged between .584 till .832 for somatic factor and between .447 till .876 for the cognitive factor. Square multiple correlation for somatic dimension of trait anxiety corresponded to, on average, .506 (.341≤SMC≤.692), and .607 for cognitive dimension (.200≤SMC≤.767). These results suggested that the factors were explaining, on average, at least half of the variance of the indicators (Table 3). The correlation between somatic and cognitive latent factors was of large magnitude (greater than .50; see [48]), specifically .674 for state model and .705 for trait model.

**Table 3:** Standardized (Unstardardized) factor loadings: State and trait models of STICSA (MS2 and MT2).

Factor	Item	State model	Trait model
Somatic	My heart beats fast	.614 (1.000)	.711 (1.000)
	My muscles are tense	.652 (1.063)	.676 (0.950)
	I feel dizzy	.736 (1.200)	.721 (1.015)
	My muscles feel weak	.692 (1.127)	.713 (1.003)
	I feel trembly and shaky	.787 (1.283)	.832 (1.170)
	My face feels hot	.546 (0.890)	.630 (0.886)
	My arms and legs feel stiff	.770 (1.256)	.793 (1.116)
	My throat feels dry	.538 (0.877)	.636 (0.895)
	My breathing is fast and shallow	.744 (1.212)	.799 (1.124)
	I have butterflies in the stomach	.676 (1.101)	.685 (0.963)
	My palms feel clammy	.486 (0.793)	.584 (0.821)
Cognitive	I feel agonized over my problems	.753 (1.000)	.828 (1.000)
	I think that others won’t approve of me	.722 (0.958)	.754 (0.911)
	I feel like I’m missing out on things because I can’t make up my mind soon enough	.710 (0.943)	.753 (0.910)
	I picture some future misfortune	.762 (1.012)	.834 (1.008)
	I can’t get some thought out of my mind	.764 (1.014)	.828 (1.001)
	I have trouble remembering things	.404 (0.536)	.447 (0.540)
	I think that the worst will happen	.814 (1.080)	.876 (1.058)
	I keep busy to avoid uncomfortable thoughts	.575 (0.763)	.700 (0.846)
	I cannot concentrate without irrelevant thoughts intruding	.756 (1.003)	.810 (0.979)
	I worry that I cannot control my thoughts as well as I would like to	.821 (1.090)	.866 (1.046)

#### Reliability analysis

The reliability of the factors was assessed by computing Omega for categorical data, with results supporting a very good reliability level. In the state dimension, the Omega was .895 for the somatic factor and .912 for the cognitive factor. In the trait dimension, the index presented values of .918 for the somatic dimension and .945 for the cognitive dimension. Cronbach alpha was also computed, allowing to compare our results with other studies. Results also supported a very good level of internal consistency. The state dimensions showed alphas of .803 for the somatic factor, and .869 for the cognitive factor, with item-total correlations of at least .35 for all the items of both factors. The trait dimensions presented an alpha of .869 for the somatic factor and .857 for the cognitive factor, with strong item-total correlations of at least .45.

#### Measurement invariance

Sex invariance was tested considering both state and trait models separately, aligned with the chosen strategy to further explore the psychometric models. In a first step, the models for male and female participants were tested separately, evidencing very good fit indexes. Although chi-square tests were significant, it was possible to assume the structure as equivalent in the two groups. The nested models were then tested sequentially adding restrictive constrains: (1) configurational, to assure that the factorial structure is equivalent across the sex groups; (2) metric, to test if the loadings are equivalent in the two groups; and (3) scalar, to test if the loadings and the intercepts are considered equal and, therefore, the scores on the two dimensions (somatic and cognitive) can be compared across sex groups. The results showed a very good level of overall adjustment, with CFI and TLI values above the cut-off of .95, and RMSEA lower than .07 in all the tested models. Although the individual results of the models were good, and even considering the slight improvements in the models with the increase in the constrains, the chi-square differences revealed that it was possible to assume configural and metric invariance, but not scalar invariance, in both trait and state forms (Table 4). The overall findings suggest that both male and females interpreted the underlying factorial structure of STICSA-State and STICSA-Trait, their loadings, but not intercepts, in an equivalent way.

**Table 4:** Fit indexes for sex invariant models (n_male_ = 400; n_female_ = 753).

Invariant Models	χ^2^	df	Δχ^2^	CFI	ΔCFI	TLI	RMSEA (90%CI)	ΔRMSEA
**State**								
Male	296.826	188		.968		.964	.038 (.030-.046)	
Female	684.423*	188		.953		.947	.059 (.054-.064)	
Configurational	951.702*	376		.959		.954	.052 (.047-.05)	
Metric	975.866*	395	42.221*	.958	.001	.956	.051 (.047-.055)	-.001
Scalar	937.195*	435	38.945	.964	.006	.965	.045 (.041-.049)	-.006
**Trait**								
Male	452.880*	188		.955		.949	.059 (.052-.066)	
Female	768.620*	188		.966		.962	.064 (.059-.069)	
Configurational	1200.481*	376		.964		.960	.062 (.058-.066)	
Metric	1245.004*	395	64.774*	.963	-.001	.961	.061 (.057-.065)	-.001
Scalar	1185.691*	435	46.939	.968	.005	.969	.055 (.051-.058)	-.006

*Note*. Statistics: Chi-square (χ^2^); Comparative fit index (CFI); Tucker and Lewis Index (TLI); Standardized Root Mean Squared Residual (SRMR); Root Mean Square Error of Approximation (RMSEA).

* p < .001.

#### Concurrent and nomological validity

Regarding concurrent validity, STICSA was highly associated with a measure of anxiety (STAI), specifically regarding its cognitive dimensions in both state and trait forms. The highest associations were observed between STICSA trait-cognitive dimension and STAI trait (r = .796), followed by the relationship between STICSA state-cognitive dimension and STAI state (r = .764), suggesting that STAI covers largely the cognitive aspects of anxiety. These correlation values are in line with the cut-off suggested by Kline [50] for concurrent validity (r < .75). On the other hand, the association between the somatic dimensions of STICSA and the dimensions of anxiety measured by STAI was lower (between .452 and .508), highlighting the distinction between cognitive and somatic dimensions.

Correlations with depression (DASS-D) were lower compared to the ones with anxiety (STAI), regarding the four dimensions of STICSA. Still, the level of association between the depression measure’s score and STICSA’s state-cognitive (r = .652) and trait-cognitive (r = .634) dimensions presented a large effect (>.50; [48]). When comparing the correlation between depression and both STAI state (r = .643) and STAI trait (r = .722) scores, STICSA dimensions evidenced lower levels of association with depression in all dimensions, with the exception of state-cognitive dimension. The difference in the correlations of the four STICSA factors and the positive and negative affect (PANAS) was about twice the level of association, being the association with positive affect small (.10≤r < .30) and with negative affect moderated (.30≤r < .50) in somatic dimensions and large in cognitive dimensions (>.50; Table 5). On the other hand, STAI evidenced moderate correlations with positive affect (STAI State: r = -.468; Trait: r = -.517), and strong correlations with negative affect (STAI State: r = .694; Trait: r = .723).

**Table 5:** Cronbach’s α and Pearson correlation between STICSA factors and anxiety (STAI), depression (DASS-D), positive affect (PA) and negative affect (NA).

	N° items	α	STICSA State Somatic	STICSA State Cognitive	STICSA Trait Somatic	STICSA Trait Cognitive
STAI state	20	.50	.508	.696	.459	.657
STAI trait	20	.45	.452	.764	.500	.796
DASS-D	7	.87	.426	.652	.393	.634
PANAS PA	10	.89	-.188	-.339	-.181	-.320
PANAS NA	10	.89	.436	.655	.466	.677

*Note*. Ns range from 1136 and 1139. All correlations are significant at p < .001.

### Discussion

The present study aimed to extend STICSA’s psychometric studies by exploring its dimensionality, measurement invariance, reliability and nomological validity. Our results replicated previous findings, providing strong evidence for STICSA’s construct validity, including the distinction between the four dimensions, in non-clinical samples [13, 17, 22]. The theoretical structure was reflected in the underlying factorial structure considering either the test with the total of items and the four dimensions (full factorial model), and the models separated by state and trait dimensions. This further corroborates the psychometric properties and validity qualities of this new anxiety measure in the Portuguese population.

Several full and state/trait factor models were tested in the present study, considering the theoretical structure of STICSA. Regarding the full model with the 42 items, in the present study, the best overall adjustment was observed for the model with four correlated factors, thus replicating the previous findings provided by studies testing the full model in non-clinical samples [17, 22]. Yet, considering the high multicollinearity observed between the state-cognitive and the trait-cognitive, as well as between the state-somatic and trait-somatic dimensions, several models with the state and trait forms analyzed separately were tested. These analyses revealed that the model with the best adjustment encompassed two correlated factors (cognitive and somatic) within each form. The correlations between somatic and cognitive dimensions were higher than .65 across state and trait forms, suggesting that, despite of being independent measures, they are closely associated [13]. Similar results were also found for non-clinical samples [13, 17–20] which, altogether, corroborates this instrument’s construct validity and the distinction between the four dimensions of anxiety (trait, state, as well as cognitive and somatic anxiety). The reliability analyses conducted in the present study also support STICSA as a reliable measure of anxiety, extending the previous literature [13, 16–19, 22].

In order to be accurate in group comparisons, individuals of different groups should interpret the scale and its components in an equivalent way. The multi-group CFA performed in the present study supported the equivalence of the underlying structures between groups regarding sex (males vs. females) for STICSA’s scale interpretation, at the level of metric invariance, which adds further strength to the quality of this measure and reinforces the confidence in comparing the latent variable scores across groups. A similar result regarding measurement invariance across sex was reported by past studies [18, 19]. The intercepts (the means) differ between the groups, which reinforces the expected sex differences [42], maintaining the good characteristics of the STICSA regarding the factorial structure and its loadings, although not its intercepts.

The level of association between the dimensions of STICSA and the two subscales of STAI gave further evidence of convergent validity, particularly regarding the cognitive dimensions. The difference in the levels of association between both cognitive (>.75) and somatic (.45 - .51) dimensions and the STAI state and trait scores highlights the newness and relevance of the somatic subscales of STICSA. Our results, in line with the previous literature [17, 18], thus suggested that STAI is a more specific measure of cognitive anxiety, while STICSA opens the possibility of evaluating anxiety in a more comprehensive fashion, i.e., in a multidimensional perspective. Nevertheless, although STICSA aims to provide a better differentiation between anxiety and depression, our results do not fully support the achievement of this goal. In fact, the Portuguese version of STICSA was shown to be strongly correlated with depression, particularly its cognitive dimensions (r>.50). These results are in line with previous psychometric studies of STICSA [17, 19], are also expected in light of the tripartite model of anxiety and depression [51]. This model explains the frequent overlap and comorbidity between anxiety and depression, and highlights a component specific to anxiety (high physiological arousal), as well as a component specific to depression (low positive affect), and a third component shared by both of them, corresponding to negative affect.

Hereupon, it is expected that anxiety and depression overlap and correlate at a certain extent, in particular considering the cognitive dimensions of STICSA, which seem to greatly reflect negative affect in comparison with the somatic dimensions which, in turn, reflect physiological arousal [16]. Furthermore, according to the tripartite model, it is not expected that a measure of anxiety encompasses items reflecting low levels of positive affect, which is exclusive to depression. Our results, once more, support both the tripartite model assumptions and STICSA as measure of anxiety, by showing that its cognitive dimensions are more associated with high negative affect (r≥.30 for somatic dimensions and r>.50 for cognitive dimensions) than with low positive affect (.10≤r < .30). Therefore, STICSA seems to better discriminate anxiety from depression in comparison with STAI, given STICSA’s weaker correlations with low positive affect, as well as the lower levels of association with the depression measure. Nevertheless, the lower associations with depression were observed for all dimensions of STICSA, except for the state-cognitive; this result is intriguing and new, and possibly suggests that the state dimension reflects negative affect in a higher extension than the trait-cognitive dimension. Importantly, these results highlight the relevance of the somatic subscales’ scores to differentiate anxiety from depression [17, 18].

## Study 2: Relationship between trait anxiety and the subjective and psychophysiological emotional response towards distinct emotional situations

Given the multidimensional nature of anxiety, it is of utmost relevance to consider, in self-report validations, how the scores are associated with such dimensions, as well as to the emotional experiences under distinct situations. Ree and colleagues [13] explored how the STICSA’s cognitive and somatic dimensions of STICSA-Trait predicted the state anxiety response in a stressful event. They observed that trait-cognitive anxiety predicted a significant part of the variance in the self-reported state-cognitive and state-somatic anxiety under a cognitive stressor. Conversely, trait-somatic anxiety was associated with the self-reported state-cognitive and state-somatic anxiety in response to a somatic stressor. These results support trait-cognitive and trait-somatic anxiety as independent constructs, as well as the usefulness of STICSA to predict the anxiety response towards different types of stressors [13]. Yet, it is critical to extend this knowledge to other emotional contexts (e.g., such as under fear or happiness emotional states), which can be related to different demands, and, therefore, with distinct subjective, cognitive, behavioral, emotional and physiological responses (for reviews see [24, 52]).

On the other hand, it would be equally helpful to understand how trait-cognitive and trait-somatic anxiety are differently associated not only with the subjective, but also with the psychophysiological dimension of the emotional response. In fact, the anxiety response is multidimensional, and is often characterized by differences in the psychophysiological domain. For instance, high self-reported trait anxiety has been associated with autonomic dysfunction, specifically with a decrease in vagally-mediated indexes of HRV [53, 54]. Reduced HRV, in turn, reflects decreased flexibility and adaptability, influencing general well-being and health [55, 56]. To date, no study has explored this relationship considering self-reported trait anxiety as measured by STICSA; its differentiation between the cognitive and somatic dimensions of anxiety can yield important implications, considering that many anxiety instruments, such as STAI, possibly cover items that mainly assess the cognitive dimensions of anxiety (as supported by our results of Study 1).

Adopting a multimodal perspective to assess the predictive validity of STICSA-Trait can add to the conceptualization of trait-cognitive and trait-somatic anxiety as distinct constructs [13], which have been shown to distinctively predict behavior [57]. Also, given the inter-individual variability found in the emotional response associated with the different components of emotions [23], a multimodal approach can contribute to better understand people’s emotional responses [58]. The current study sought to explore how STICSA-Trait explains differences in psychophysiological and self-report measures. We designed an emotional induction procedure, during which the subjective and psychophysiological responses of the participants were collected. We expected to observe an overall reduced HRV in individuals with high trait anxiety in comparison with individuals with low trait anxiety [53].

### Materials and method

#### Participants

Seventy-six participants from a Portuguese university volunteered to participate in the study. Only participants with normal or corrected-to-normal visual acuity and free of any medication or disease that could influence the cardiac functioning (e.g., tricyclic antidepressants or cardiac arrhythmia) were included. Also, participants did not report any diagnosis of mental or neurological illness. All participants completed a sociodemographic questionnaire online with relevant information for the study, as well as the STICSA-Trait developed in Study 1. Two participants were later excluded for being extreme cases regarding the Low Frequency (LF) and High Frequency (HF) power values. The final sample was constituted by 74 participants, 49 females (66.2%) and 25 males (33.8%), aged between 18 and 31 years old (M = 21.41; SD = 3.09). The study was approved by the Ethics and Deontology Committee of the University of Aveiro (ref. 10/2017) and followed the same ethical procedures as in Study 1. Participants were rewarded for their participation through a draw of three 20€ vouchers.

#### Materials

*Visual Stimuli*. An emotional induction paradigm involving the visualization of film clips was used (e.g., [59]). Stimuli consisted in three sets of 8–12 film clips, with approximately 30 minutes of duration (per set). These clips were taken from horror movies, comedy movies and documentaries, to induce fear, happiness and a neutral emotional state, respectively. Moreover, three documentary clips, of approximately 5 minutes, were also selected and presented before each set of emotional videos, to provide baseline psychophysiological data. All film clips demonstrated previously their ability to induce the expected emotions [60–62].

*Evaluation of the subjective emotional response*. Three Visual Analogue Scales (VAS) of 100 points were used to assess participants’ emotional state before and after the emotional induction, covering the measurement of subjective Happiness and Fear (Emotion VAS; “How do you feel right now?”), as well as of self-reported arousal (Arousal VAS; “How much aroused did you feel during the visualizations of the clips?”), after the baseline videos and after the emotional videos. The experimental task, which included the presentation of the VAS and the videos, was programmed with the software OpenSesame (version 3.2.1; OpenSesame Inc.), using Python language (version 2.7.13). The task was performed using a Dell OptiPlex 7040 and a 17-inch Dell digital monitor (Model: E178FP), with a refresh rate of 60 Hz.

*Cardiac signal recording*. The cardiac signal was recorded using BIOPAC MP160 data acquisition system and AcqKnowledge 5 software (BIOPAC Systems, Inc.), with a sampling rate of 1000 Hz [63]. Ag/AgCl disposable vinil electrodes (EL503; BIOPAC Systems, Inc.) and conductive gel were used to acquire the signal, following a *Lead II* configuration [63].

#### Procedure

Before data collection, all participants received information about the study’s procedure and provided written informed consent. Furthermore, the experimenter asked participants beforehand if they had been through any emotionally intense situation in the past days, to assure that the sessions would be scheduled in a “emotionally neutral” period. The three sessions were spaced for at least one week, to avoid emotional contagion between sessions. The order of presentation of each emotional condition was counterbalanced between participants. Each session started with the electrodes’ placement. Following this step and a 10-minute interval to allow signal stabilization, participants were instructed to seat in a chair and place their chin on a chin rest located at approximately 60 cm from a computer monitor. Participants started the task by answering the Emotion VAS, presented in a randomized order. Then, the baseline video was presented, followed by the Arousal VAS and, finally, by the film clips (presented consecutively). In the end of the video set, a new Arousal VAS was presented, followed by the Emotion VAS. Each experimental session lasted approximately 70 minutes. The study was conducted in a quiet and well-ventilated room.

#### Design and statistical analyses

HRV was calculated considering the RR or inter-beat intervals and, in this case, several spectral components may be used to interpret the signal [56]. Three HRV indexes were considered: The High Frequency (HF; in ms^2^) band, the Low Frequency (LF; in ms^2^) band and the ratio of LF to HF power (LF/HF). The LF band encompasses frequencies varying between 0.04 and 0.15Hz and reflects both sympathetic and parasympathetic activation, as well as baroreflex mechanisms [55]. The HF band encompasses the signal power in the range from 0.15 to 0.4 Hz, and has been suggested to mainly reflect parasympathetic activity or vagal cardiac control [55, 56]. Low HF power has been, therefore, associated with situations of anxiety and stress [53]. This band also reflects variations in the cardiac signal related to the respiratory cycle [55]. Therefore, alterations in the respiration rhythm are able to significantly modify HF power, and can also influence LF values under certain circumstances (e.g., during slow respiration rates; [56]). For this reason, in this study, the chosen filters to apply on the cardiac signal were designed in order to attenuate this physiological interference. The LF/HF ratio has been suggested to reflect the autonomic balance between sympathetic and parasympathetic activity, at least under certain conditions [56]. Higher values can indicate greater sympathetic activation in comparison with parasympathetic activity depending on the context; for instance, in challenging situations requiring increased SNS activation [55].

For details about the cardiac signal processing and the HRV indexes’ calculation, please refer to S1 Fig in Supporting Information (SI). Separated repeated measures analysis of variance (ANOVA) were performed considering the psychophysiological and the self-report measures as dependent variables (LF/HF/Ratio/Happiness/Fear/Arousal), with Condition (Fear/Happy/Neutral emotional induction) and Moment (baseline/emotion) as within-subjects measures, and Group (high/low anxiety) as between-subjects measure. All the analyses were performed for groups divided considering trait-cognitive or trait-somatic anxiety. Whenever sphericity was not assumed, the values for main effects were reported using the Greenhouse-Geisser correction. Simple-effect analyses were performed for all the significant interactions, to clarify the direction of the effect. The significance levels of the comparisons were corrected using the Bonferroni correction [47]. These procedures were conducted using IBM SPSS Statistics (version 23).

### Results

#### Descriptive and preliminary analyses

Participants were divided in two groups considering the median of the cognitive dimension of STICSA-Trait (Mdn = 19): the group with low trait-cognitive anxiety (LowCG; N = 41) and the group with high trait-cognitive anxiety (HighCG; N = 33). Similarly, two groups of trait-somatic anxiety were created considering the median (Mdn = 16): the group of low trait-somatic anxiety (LowSG; N = 42) and the group of high trait-somatic anxiety (HighSG; N = 32). Descriptive statistics regarding the psychophysiological and subjective measures for each condition and moment of evaluation considering the four groups are outlined in S1 and S2 Tables (SI). Before proceeding to the main analyses, differences across baselines were also tested to assure no significant differences in the dependent variables at the beginning of each condition. Results of repeated measures ANOVAs revealed no differences between baselines across measures, except regarding the evaluation of happiness, F(1.790) = 3.352, p = .043, partial η^2^ = .044. Nevertheless, the post-hoc tests were all non-significant.

Furthermore, we tested if the groups of cognitive and somatic anxiety were equivalent in the pre-test, considering the three emotional conditions and for all measures. Results of repeated measures ANOVAs suggested no differences between groups across conditions and measures, except regarding LF/HF ratio (trait-somatic anxiety groups) and self-reported happiness (trait-cognitive anxiety groups). In this line, there was a main effect of Condition regarding self-reported happiness, only when considering the groups of trait-cognitive anxiety, F(1.793) = 3.536, p = .037, partial η^2^ = .074. Nevertheless, the post-hoc tests were all non-significant. Also, there was a significant main effect of Group (groups of trait-somatic anxiety) regarding the LF/HF ratio, F(1) = 7.513, p = .008, partial η^2^ = .094, suggesting that the HighSG had significantly higher LF/HF ratio than the LowSG in the baseline moments. Yet, the interaction Condition x Group was non-significant (p = .674), suggesting that this group difference was systematically observed across conditions and is most likely due to the group characteristics (i.e., higher levels of self-reported trait-somatic anxiety may be associated with higher LF/HF ratio at rest, in comparison with lower levels of trait-somatic anxiety). For the detailed results of these preliminary analyses, please refer to S3 and S4 Tables (SI). Altogether, these results indicate that the equivalence of the baselines can be assumed.

#### Differences considering groups of trait-cognitive anxiety

**Fig 1:**
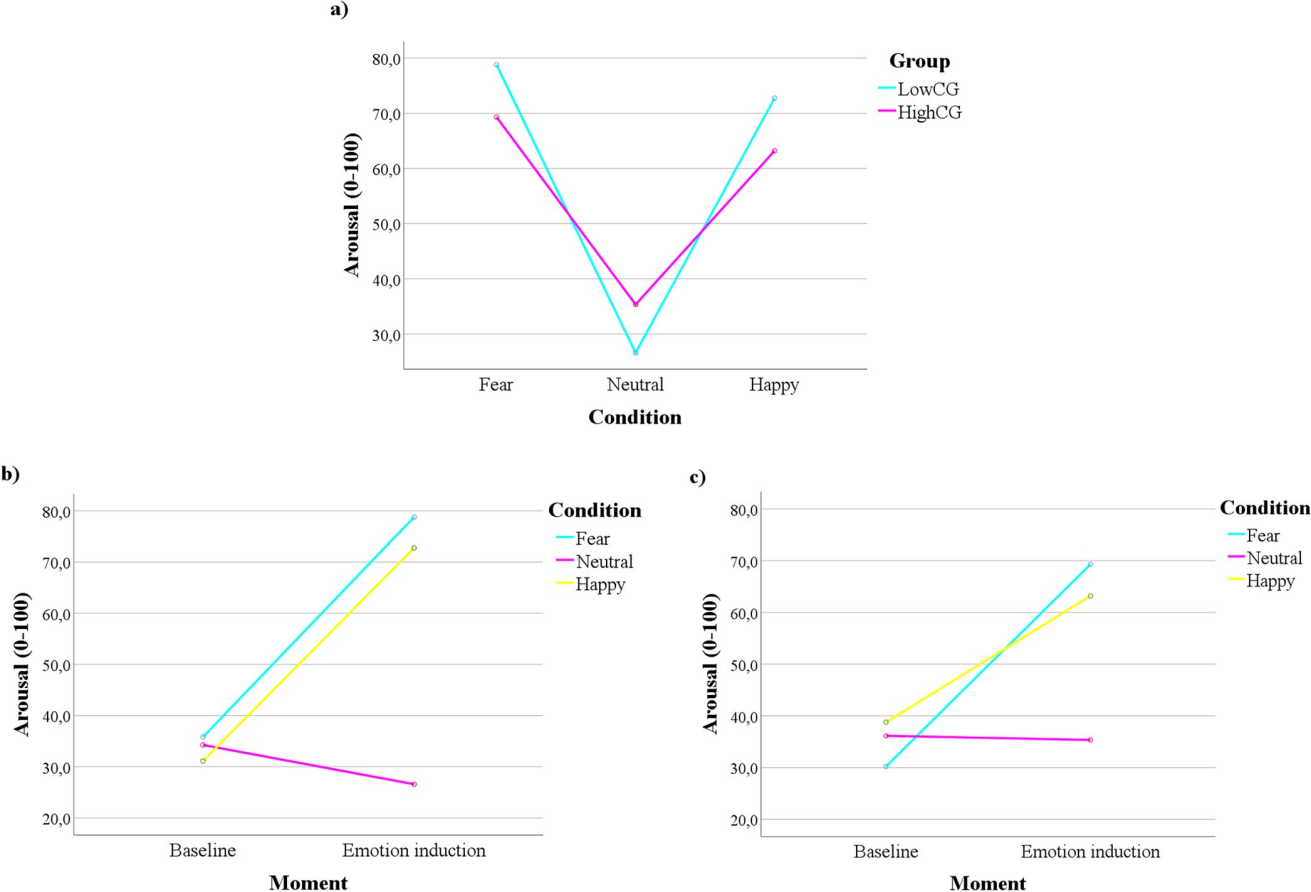
Cognitive groups’ differences regarding the self-reported arousal. a) Group differences in the self-reported arousal after the emotional induction; b) LowCG self-reported arousal across Moment and Condition; c) HighCG self-reported arousal across Moment and Condition.

For an overview of the general results of the ANOVAs regarding the trait-cognitive anxiety groups, please refer to S5 and S6 Tables (SI). Considering the psychophysiological measures, there was not a significant main or interaction effect of Group. However, in the self-report measures, a significant third order interaction effect Condition x Moment x Group emerged, F(2) = 3.414, p = .036, partial ƞ^2^ = .045. The simple-effect analysis suggested that, in the neutral emotional condition, the LowCG reported higher levels of arousal in the baseline in comparison with the emotional condition, p = .025. Yet, the HighCG did not present significant differences between the baseline and the emotional condition, in the self-reported arousal, p = .833. In the other conditions, the performance was similar in both groups. Additionally, when analyzing the graph illustrating group differences across emotional conditions (Fig 1A), it is possible to observe that, in the LowCG, the self-reported arousal seemed to vary more across conditions, in comparison with the HighCG. Similarly, the variation between the baseline and the emotional conditions seems to be more consistent and marked in the LowCG in comparison with the HighCG, especially for the happy and neutral conditions (Fig 1B and 1C).

#### Differences considering groups of trait-somatic anxiety

**Fig 2:**
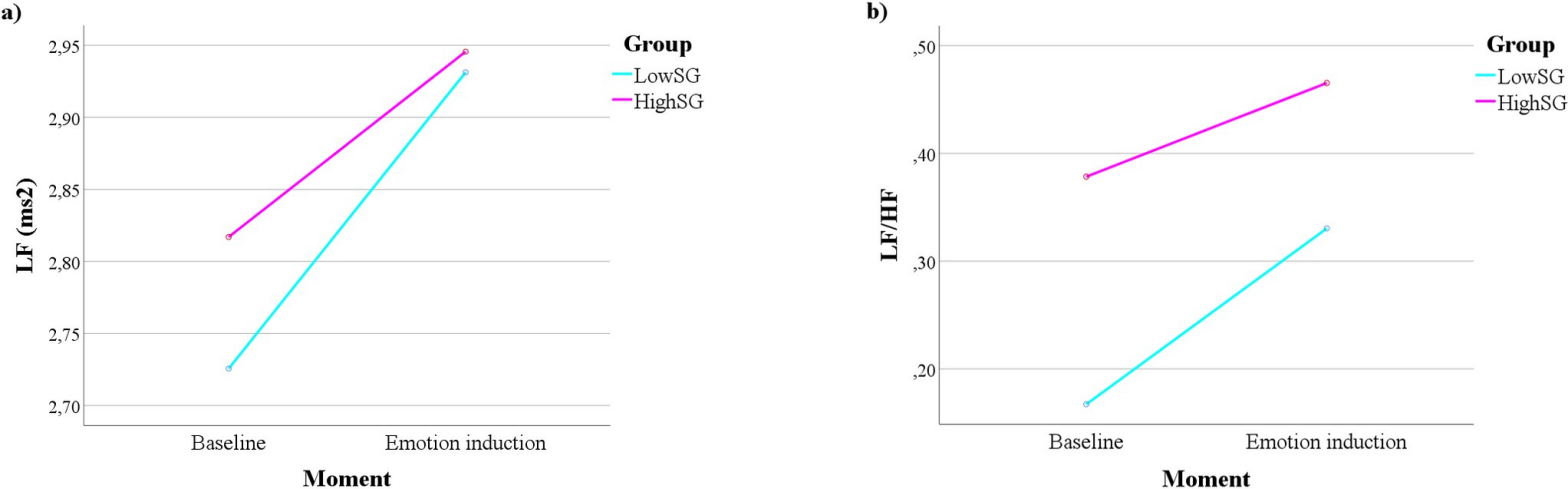
Somatic groups’ differences considering the moment of evaluation. a) LF power and b) LF/HF ratio.

For an overview of the general results of the ANOVAs considering the trait-somatic anxiety groups, please refer to S7 and S8 Tables (SI). A significant interaction Moment x Group emerged regarding the LF power, F(1) = 5.132, p = .026, partial ƞ^2^ = .067. The simple-effects inspection suggested that the LF power was always lower in the baselines in comparison with the emotional conditions, p < .001. Additionally, there were no significant differences between groups neither at the baselines nor at the emotional conditions. When inspecting the graph (Fig 2A), the HighSG seems to have slightly higher LF power in the baseline evaluation in comparison with the LowSG; however, the difference was small and not significant. Furthermore, a significant main effect of Group emerged regarding the LF/HF ratio, F(1) = 6.390, p = .014, partial ƞ^2^ = .082, suggesting that the HighSG presented higher values of LF/HF in comparison with the LowSG. Likewise, there was a significant interaction Moment x Group regarding the LF/HF ratio, F(1) = 6.765, p = .011, partial ƞ^2^ = .086, suggesting that the HighSG had a significantly higher LF/HF score in baseline and emotion evaluation, in comparison with the LowSG. Furthermore, the baselines and emotion evaluation were always significantly different in both groups. The analysis of the graph (Fig 2B) reinforced that the differences in the ratio score between the baseline and emotion evaluation were slightly less accentuated in the HighSG compared to the LowSG.

**Fig 3:**
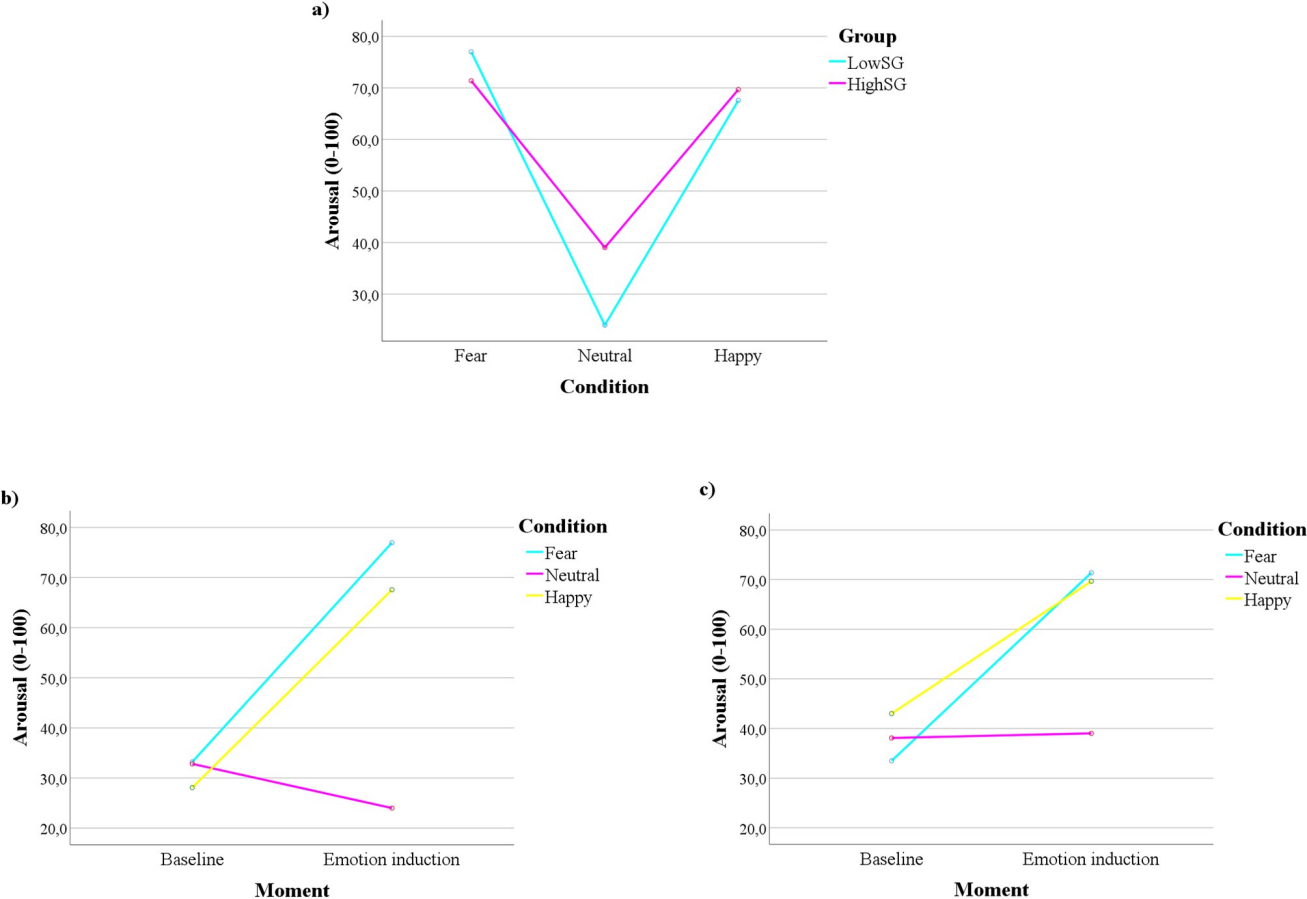
Somatic groups’ differences regarding the self-reported arousal. a) Group differences in the self-reported arousal after the emotional induction; b) LowSG self-reported arousal across Moment and Condition; c) HighSG self-reported arousal across Moment and Condition.

Regarding the self-report measures, we observed a marginally significant interaction Condition x Group, F(2) = 3.053, p = .05, partial ƞ^2^ = .041, suggesting that, in both groups, the self-reported arousal was always higher in the fear condition compared with the neutral condition, as well as in the happy condition compared with the neutral condition, p < .01. There were no significant differences between groups across the three emotional conditions. Importantly, a significant interaction Condition x Moment x Group emerged, F(2) = 3.102, p = .048, partial ƞ^2^ = .048. The post-hoc tests suggested that, while in fear and happiness conditions there were always significant differences between the self-reported arousal in the pre- and post-emotional induction in both groups, p < .001, in the neutral condition there were no significant differences between the self-reported arousal in the pre- and the post-emotional induction for the HighSG, p = .805. Moreover, after the neutral condition, the HighSG reported significantly more arousal than the LowSG, p = .015 (see Fig 3A). The analysis of the graphs allows to further observe that, in the neutral condition, while there is a slight decrease in the self-reported arousal in the post-induction in comparison with the pre-induction in the LowSG (Fig 3B), in the HighSG (Fig 3C) the values of self-reported arousal pre- and post-induction are quite similar. Also, the LowSG seems to have a higher difference between the self-reported arousal after the fear condition and the self-reported arousal after the happiness condition, while in the HighSG this difference is narrower. Lastly, the post-hoc tests suggest that, before the happiness induction, the HighSG also reported significantly more arousal than the LowSG, p = .013.

### Discussion

In a previous study, STICSA-Trait cognitive and somatic dimensions were found to predict state anxiety towards cognitive or somatic stressors, respectively [13]. The present study sought to extend the knowledge about STICSA-Trait’s predictive validity, by examining the performance of groups of high and low trait-somatic/cognitive anxiety considering the psychophysiological and subjective dimensions of the emotional response in three distinct laboratory-induced emotional situations. Results showed that the trait-cognitive anxiety dimension, as measured by STICSA, was related to differences between groups regarding self-reported arousal. Conversely, the trait-somatic anxiety dimension was observed to be associated with differences between groups regarding psychophysiological measures, specifically the LF and LF/HF indexes of HRV, as well as with differences regarding self-reported arousal. These results highlight the relevance of STICSA-Trait as a measure that assesses different aspects of anxiety, distinctively associated with different components of the emotional response.

Considering the groups divided by trait-cognitive anxiety, our analysis suggested that the LowCG reported significantly more arousal in the baseline than in the emotionally neutral condition. This result suggests that the emotionally neutral condition contributed to a decrease in the experienced arousal in people with low trait-cognitive anxiety. However, people with high trait-cognitive anxiety reported constant levels of arousal, considering the transition from the baseline to the neutral condition. This may reflect the experience of increased physiological activation often reported by patients with anxiety disorders [64]. Visual inspection of the data further suggested that the self-reported arousal varied more across emotional conditions in the LowCG in comparison with the HighCG. Furthermore, variations between the baselines and each respective emotional condition seemed to be more consistent and noticeable in the LowCG in comparison with the HighCG. Together, these results suggest a relationship between lower emotional flexibility and high self-reported trait-cognitive anxiety, particularly considering the emotionally neutral or non-aversive conditions. This is consistent with the literature that observed lower variability regarding self-reported arousal in the context of anxiety disorders (e.g., [65]). Nevertheless, these differences should be interpreted with caution, since they were non-significant; moreover, our sample is non-clinical, limiting direct comparisons.

Interestingly, the results considering the trait-cognitive anxiety groups suggested no differences regarding the psychophysiological measures. This suggests that the cognitive dimension of STICSA-Trait is more related to self-report, specifically the self-reported arousal, and is not related to differences in any of the indexes of HRV. This also highlights the frequently observed weak relationship between the self-report and physiology dimensions of emotion [23], and particularly the frequently observed incoherence between heightened self-reported arousal and unchanged physiology in anxiety disorders [65, 66]. This particular relationship with the cognitive dimension of trait anxiety may reflect a set of cognitive factors that lead to an altered attention and perception of the self-arousal levels, inconsistent with the physiological changes [67]. Yet, although the pattern of self-reported arousal was different between the baseline and emotionally neutral condition within each group of low/high trait-cognitive anxiety, and the self-reported arousal after the emotionally neutral condition was numerically higher in the HighCG, there were no significant differences between groups in the post evaluation; the significant results were only observed when considering the differences between the baseline and the emotionally neutral condition within each group.

Results regarding the groups divided by trait-somatic anxiety suggested that there were no differences within each moment of evaluation, neither differences between pre- and post-emotional induction within groups, considering the LF component of HRV. Even so, after visually inspecting data, it was possible to observe that the HighSG presented higher LF power in the baseline condition, in comparison with the LowSG (non-significant). The results regarding the LF/HF ratio add to the interpretation of this interaction; in fact, we also observed that the HighSG presented higher LF/HF values in comparison with the LowSG, both in the baseline and during the emotional conditions. By visually inspecting data, differences between the baseline and emotion conditions seemed to be lower in the HighSG in comparison with the LowSG, since the former started with a higher LF/HF score at the baseline, and thus had a less accentuated rise during the emotional conditions. Together, these results may suggest that self-reported trait-somatic anxiety is related to objective physiological alterations in healthy adults. Consistently with the previous literature, these alterations may be related to the often observed reduced physiological flexibility [64] and abnormal ANS activity in anxiety [68].

Considering that the ratio LF/HF has been suggested to reflect sympathovagal balance, with higher values associated with sympathetic dominance [56], our findings may suggest that self-reported trait-somatic anxiety is associated with elevated sympathetic activation, particularly at rest and in non-threatening emotional situations. These results are not in accordance with the literature concerning healthy individuals with high levels of trait anxiety, which has been reporting reduced HF power in high trait anxiety [53, 54, 69, 70]. Nevertheless, some studies have observed greater sympathetic activity in patients with anxiety disorders in comparison with control groups at resting conditions, as suggested by higher LF power, higher LF/HF ratio or both [71, 72]. However, a meta-analysis conducted by Chalmers and colleagues [73] suggested that anxiety disorders do not have a significant impact in LF values, with the effects in HRV being more evident through differences in the HF band.

It is important to note that several aspects may contribute to differences in the HRV scores found across studies. These include variables associated with the recording, such as the methods employed to control for artifacts and respiration confounds, as well as variables associated with the subjects, including age and health status [56, 74]. Importantly in the context of our results, the tasks used for the recording may add important differences that also preclude comparisons. In fact, previous studies have explored the relationship between HRV and trait anxiety solely at rest conditions [54, 69, 70] or during relaxation and mental stress conditions [53]. Some of these studies have also analyzed only certain indexes of the ANS activity, such as vagally mediated components of HRV [54, 69], which does not allow to draw conclusions about the other measures.

Similarly to the observed in trait-cognitive anxiety groups, our results also suggested that trait-somatic anxiety is associated with self-reported arousal–which would be expected, since self-reported arousal include the subjective experience of physiological activation. Specifically, group differences emerged after the neutral induction, where the HighSG evinced higher levels of self-reported arousal in comparison with the LowSG. By inspecting data visually, while the levels of self-reported arousal seemed to decrease from the pre-induction to the post-induction in the LowSG, in the HighSG the levels of self-reported arousal remained constant. Once more, these results point to increased self-reported physiological activation and lower variability regarding self-reported arousal in people with higher levels of anxiety [64, 65].

Although our results contribute to unravel the association between trait-cognitive/somatic anxiety and different dimensions of the emotional response towards emotional situations, some factors limit the conclusions to draw. For instance, some authors have been claiming that the ratio LF/HF, which has been often addressed as an indicator of sympathovagal balance, should be interpreted with caution, especially in short-length recordings and considering the specific recording conditions (e.g., if it is recorded in a resting condition or not, if it includes paced or normal breathing; [55, 56]). In fact, the LF component of HRV is not a pure index of sympathetic activity [55]. Moreover, respiration can highly influence both the LF and HF parameters [56], thereby, socioemotional tasks that are associated with changes in the respiratory pattern have an impact in these HRV parameters (for a review about this issue see [74]). In the present study, the respiratory pace was not explicitly controlled since it would affect the emotional response. Therefore, caution is needed when interpreting these HRV indexes, and future studies should further investigate the relationship between trait anxiety and variations in the different HRV parameters.

Another limitation of the present study concerns the relatively small sample size, which impacts statistical power. Furthermore, in lower sample sizes, small variations may conduct to group differences; this may explain why we observed trait-somatic anxiety group differences regarding self-reported arousal in the pre-induction moment of the happiness condition when analyzing the statistically significant Condition x Moment x Group interaction. In the preliminary analyses (see the “Descriptive and preliminary analyses” section of Results, where we performed Condition x Group repeated measures ANOVA), we did not find statistically significant group differences regarding self-reported arousal in the pre-induction moment across conditions. For this reason, we assumed that the groups have started from the same point across conditions. Yet, our results should be interpreted with caution and future studies should replicate these findings with larger samples. Finally, it is also important to note that participants’ emotional response was elicited and evaluated following laboratory-induced emotions, which may have resulted in “artificial” emotional responses, differing in intensity and valence from the observed emotional responses in the daily life [59, 75]. Therefore, future studies should extend this research by assessing emotional response considering its subjective, psychophysiological, and behavioral components in more ecological contexts, using less invasive and limiting procedures, in order to closer understand the relationship between self-reported anxiety and emotional response across daily situations.

## General discussion

The present study aimed to extend STICSA’s validation studies to the Portuguese context, through several analyses of dimensionality, measurement invariance, reliability and nomological and predictive validity. Results supported STICSA as a useful instrument measuring cognitive and somatic symptomatology dimensions within state and trait anxiety. The first study provided evidence for excellent psychometric properties and replicated previous findings by supporting the instrument’s construct validity, with its four dimensions structure and with separate structures for trait and state anxiety. Furthermore, it supported the equivalence of measure across groups of sex when considering the factor structure and loadings (metric invariance). STICSA also evidenced good nomological validity, despite of being strongly correlated with depression, particularly its cognitive dimensions. Our results also suggested that STICSA is possibly a better instrument to differentiate anxiety from depression, which constitutes an important improvement in the context of anxiety assessment. The second study further highlighted the relationship between trait-somatic anxiety and differences in both the subjective and psychophysiological domains of the emotional response, as well as the association between the trait-cognitive anxiety and the subjective experience of arousal. The ability to provide a better differentiation between anxiety and depression, allied to excellent psychometric properties and a broader scope of evaluated symptoms, as well as to the knowledge about the distinct relationships between the cognitive and somatic dimensions and the subjective and psychophysiological components of the emotional response, makes STICSA a privileged source of information with the potential to critically shape a targeted assessment, monitoring and intervention.

Although the present study adds important evidence about the utility and validity of STICSA, some considerations about the conclusions to draw should be addressed. First, our results are restricted to the general population. It would be important to assess STICSA’s utility, as well as the generalizability of the present results to other populations where anxiety may have distinct manifestations, such as in healthy older adults [22] and in clinical populations [16]. Extending this research to clinical settings may be particularly critical to acknowledge the equivalence of measure between clinical and non-clinical populations and/or to evaluate the association with other psychopathological measures. Until date, only two studies investigated STICSA’s properties in clinical samples [16, 76]; moreover, apart from the present research, there is only one more study assessing the predictive validity of STICSA-Trait, also in a non-clinical population [13]. Thus, future studies are needed to expand this knowledge and establish cut-off values for the four dimensions of STICSA, considering the Portuguese clinical population. Moreover, more than half of our sample was constituted by students (57.2%), which may limit the generalization of our results; future studies should extend STICSA’s validation studies by recruiting more diversified samples of the general population. Lastly, caution is needed when directly comparing our results with the results obtained by other psychometric studies of STICSA, since distinct studies have been using different response labels, specifically considering the STICSA-Trait form. In fact, Ree and colleagues [13] described that the STICSA-State form should be scored from 1 (not at all) to 4 (very much), whereas the STICSA-Trait form should be scored from 1 (almost never) to 4 (almost always). This response scale, which differentiates the two STICSA forms in terms of both the response labels and instructions, was subsequently used by other researchers [18, 19]. However, similarly to the indicated in the work of Grös and colleagues [16], which was the first published psychometric study of STICSA, several other authors have been using the same response labels for both the STICSA-State and STICSA-Trait forms (i.e., both forms ranging from 1 [not at all] to 4 [very much]; [17, 20, 22]). In the present study, we followed the response labels provided by Grös and colleagues [16], which could have made it more difficult to distinguish between the state and the trait forms, since only the instructions differed. Yet, it is important to note that the studies, in general, have been supporting similar results in terms of STICSA dimensionality, reliability and nomological validity, which suggests that this difference in the response labels do not substantively affects the results.

Notwithstanding the limitations discussed throughout the article, important strengths are worth to note. First of all, an extensive psychometric evaluation was performed, including also a predictive validity analysis of STICSA-Trait, which is an analysis that is generally lacking in the literature concerning instruments’ validation and can add very important information about the usefulness of an instrument in specific contexts. Moreover, we have assessed STICSA’s predictive validity considering different emotional contexts (happy, fear and neutral). Most studies assess the relationship between anxiety and emotional response in neutral (at rest; e.g., [54]) or under negative emotional inductions (such as stress-inducing situations; [13, 53]). Although these studies give valuable information about an individual’s response, their results are limited to those contexts in particular. Thereby, it is important to extend this research and measure emotional response in other contexts that pose different demands and are associated with distinct responses (e.g., such as situations that induce positive emotions; [24, 52]), in order to have a broader comprehension of how self-reported anxiety predicts an individual’s behavior. Finally, we assessed STICSA’s predictive validity by adopting a multidimensional approach of the emotional response, which gives important hints about the relationship between the self-reported anxiety (considering distinct dimensions) and its association with different aspects of the emotional response. This approach further allowed the analysis of the coherence among response systems (which is often low; [23, 65]), as well as of the relationship between STICSA and HRV, a biomarker of autonomic dysfunction and psychopathology [55].

## Conclusion

The present research supported STICSA as an adequate instrument to measure anxiety symptoms in the Portuguese population, by extending its psychometric studies and reinforcing it as a multidimensional and robust measure of anxiety in its four dimensions: state-cognitive, state-somatic, trait-cognitive and trait-somatic. By embracing a greater scope of symptoms, STICSA has the potential to meet the demands posed by the increasing prevalence of anxiety disorders and the multiplicity of symptom profiles manifested by different individuals in different contexts. Furthermore, considering the association between STICSA-Trait and the psychophysiological and subjective dimensions of the emotional response, this instrument may provide additional valuable information about how individuals would feel, think and respond in specific situations [13], improving the knowledge about their functioning, and leading to the development of a more appropriated evaluation, monitoring and intervention plans.

## Supporting information

S1 Fig Cardiac signal processing and calculation of the HRV indexes. (DOCX)

S1 Table Means and standard deviations regarding psychophysiological and self-report measures, considering trait-cognitive anxiety groups. (DOCX)

S2 Table Means and standard deviations regarding psychophysiological and self-report measures, considering trait-somatic anxiety groups. (DOCX)

S3 Table Results of the repeated measures ANOVA, regarding differences between the baselines of the three emotional conditions. (DOCX)

S4 Table Results of the repeated measures ANOVA, regarding differences between groups considering the three emotional conditions at the baseline moment. (DOCX)

S5 Table ANOVA’s results regarding the psychophysiological measures, considering trait-cognitive anxiety groups. (DOCX)

S6 Table ANOVA’s results regarding the self-report measures, considering trait-cognitive anxiety groups. (DOCX)

S7 Table ANOVA’s results regarding the psychophysiological measures, considering trait-somatic anxiety groups. (DOCX)

S8 Table ANOVA’s results regarding the self-report measures, considering trait-somatic anxiety groups. (DOCX)

S1 Database Database study 1. (CSV)

S2 Database Database study 2. (CSV)
