# Supplementary material for: Multidimensional assessment of anxiety through the State-Trait Inventory for Cognitive and Somatic Anxiety (STICSA): From dimensionality to response prediction across emotional contexts
Source: PLoS One. 2022 Jan 25;17(1):e0262960. doi: 10.1371/journal.pone.0262960 (PMC8789173; doi:10.1371/journal.pone.0262960)
Supplement: S1 Fig — (DOCX) [file pone.0262960.s001.docx]

**S1 Fig. Cardiac signal processing and calculation of the HRV indexes.**

**Cardiac signal processing**

- Considering that the HRV evaluates the variation inherent to inter-beat intervals, the first step on the algorithm was the R-peak detection.
  - R-peaks were detected using a widely known algorithm (Kathirvel et al., 2011), with a Random Sample Consensus (RANSAC) window size of 5.
  - The cut-off frequencies of the band pass filter were 5 Hz and 15 Hz, respectively.
- The RR intervals were obtained by the consecutive differences between two R-peaks.
- A pre-processing step was performed to guarantee robustness of the data; in this step, outliers were removed by ignoring R-R differences below 300 ms or above 2000 ms, according to the best practices described by Inbar and colleagues (1994).
- Furthermore, ectopic beats were removed using the process described by Kamath and colleagues (1995), with the Malik method (Malik, 1995), considering a removing rule of 0.2.

**Calculation of the HRV indexes**

- The HRV metrics were calculated over the normal-to-normal beats.
- All the frequency domain features were computed using the Welch method, considering a sampling frequency of 7 Hz, a linear interpolation, an LF band between 0.04 Hz and 0.15 Hz and a HF band between 0.15 Hz and 0.40 Hz.
  - This process was performed both in the baseline cardiac signal, and in the signal recording during emotional induction.
- The chosen HRV metrics were calculated over a window of at least 5 minutes of duration to allow the method to infer the individual variability; the analysed baseline had 5 minutes of duration, and the emotional segment of cardiac signal had approximately 30 minutes of duration.
  - **Note:** The duration of the segments may influence the evaluation and limit comparisons between segments with distinct durations, since the information and its variability may be affected by external events. Nevertheless, in this study all the emotional segment (30 minutes) was used to describe the full emotional process.
- The code underlying all these analyses was implemented in Python 3.6 using the numpy and hrvanalysis libraries. Finally, and as expected (e.g., Castaldo et al., 2015), the HRV measures did not meet the assumption of normality and were thus log-transformed (log10) after inspecting skewness and kurtosis.

Castaldo, R., Melillo, P., Bracale, U., Caserta, M., Triassi, M., & Pecchia, L. (2015). Acute mental stress assessment via short term HRV analysis in healthy adults: A systematic review with meta-analysis. Biomedical Signal Processing and Control, 18, 370–377. https://doi.org/10.1016/j.bspc.2015.02.012

Inbar, O., Oren, A., Scheinowitz, M., Rotstein, A., Dlin, R., & Casaburi, R. (1994). Normal cardiopulmonary responses during incremental exercise in 20- to 70-yr-old men. Medicine & Science in Sports & Exercise, 26(5), 538–546. https://doi.org/10.1249/00005768-199405000-00003

**References**

Kamath, M. V., & Fallen, E. L. (1995). Correction of heart rate variability signal for ectopics and missing beats. In M. Malik & A. J. Camm (Eds.), Heart Rate Variability (pp. 75–85). Futura.

Kathirvel, P., Manikandan, M. S., Prasanna, S. R. M., & Soman, K. P. (2011). An efficient R-peak detection based on new nonlinear transformation and first-order gaussian differentiator. Cardiovascular Engineering and Technology, 2(4), 408–425. https://doi.org/10.1007/s13239-011-0065-3

Malik, M. (1995). Geometric methods for heart rate variability assessment. In M. Malik & A. J. Camm (Eds.), Heart Rate Variability. Futura.
