# Supplementary material for: Multidimensional assessment of anxiety through the State-Trait Inventory for Cognitive and Somatic Anxiety (STICSA): From dimensionality to response prediction across emotional contexts
Source: PLoS One. 2022 Jan 25;17(1):e0262960. doi: 10.1371/journal.pone.0262960 (PMC8789173; doi:10.1371/journal.pone.0262960)
Supplement: S1 Table — (DOCX) [file pone.0262960.s002.docx]

**S2 Table. Means and standard deviations regarding psychophysiological and self-report measures, considering trait-cognitive anxiety groups.**

|  |  | LowCG (N=41) | | | | HighCG (N=33) | | | |
| --- | --- | --- | --- | --- | --- | --- | --- | --- | --- |
|  |  | Baseline | | Emotion | | Baseline | | Emotion | |
| Measure | Condition | M | SD | M | SD | M | SD | M | SD |
| LF | Fear | 823.94 | 705.07 | 930.15 | 539.20 | 830.47 | 702.73 | 941.69 | 577.65 |
|  | Neutral | 744.99 | 427.57 | 994.43 | 566.85 | 772.22 | 608.58 | 1132.24 | 729.42 |
|  | Happy | 719.11 | 428.33 | 1139.23 | 612.66 | 741.16 | 700.67 | 994.72 | 601.99 |
| HF | Fear | 537.49 | 648.04 | 525.83 | 506.13 | 464.42 | 390.11 | 444.93 | 318.18 |
|  | Neutral | 466.94 | 351.60 | 428.10 | 294.90 | 483.57 | 395.87 | 540.17 | 489.04 |
|  | Happy | 545.98 | 520.77 | 556.30 | 445.94 | 416.45 | 339.60 | 425.30 | 340.50 |
| LF/HF | Fear | 2.77 | 2.82 | 2.93 | 2.43 | 2.67 | 1.82 | 2.76 | 1.63 |
|  | Neutral | 2.30 | 1.53 | 3.15 | 1.99 | 2.52 | 2.11 | 3.07 | 1.81 |
|  | Happy | 2.54 | 2.29 | 3.16 | 2.02 | 2.36 | 1.65 | 3.12 | 1.73 |
| Happy | Fear | 50.66 | 25.33 | 25.93 | 25.17 | 46.33 | 19.21 | 27.67 | 25.08 |
|  | Neutral | 51.79 | 23.72 | 45.31 | 26.97 | 46.84 | 21.66 | 45.94 | 25.57 |
|  | Happy | 47.62 | 25.62 | 73.84 | 19.22 | 39.21 | 24.95 | 66.19 | 20.03 |
| Fear | Fear | 5.20 | 13.79 | 37.99 | 34.04 | 6.88 | 13.62 | 37.76 | 31.40 |
|  | Neutral | 4.40 | 14.13 | 0.44 | 1.72 | 11.72 | 16.64 | 4.99 | 10.88 |
|  | Happy | 5.76 | 15.55 | 0.34 | 0.88 | 7.34 | 13.98 | 3.47 | 9.61 |
| Arousal | Fear | 35.82 | 30.67 | 78.78 | 23.10 | 30.22 | 25.54 | 69.30 | 27.18 |
|  | Neutral | 34.25 | 28.40 | 26.59 | 24.75 | 36.14 | 26.71 | 35.35 | 28.52 |
|  | Happy | 31.12 | 25.39 | 72.74 | 20.33 | 38.78 | 26.35 | 63.19 | 25.73 |
