# Supplementary material for: Multidimensional assessment of anxiety through the State-Trait Inventory for Cognitive and Somatic Anxiety (STICSA): From dimensionality to response prediction across emotional contexts
Source: PLoS One. 2022 Jan 25;17(1):e0262960. doi: 10.1371/journal.pone.0262960 (PMC8789173; doi:10.1371/journal.pone.0262960)
Supplement: S2 Table — (DOCX) [file pone.0262960.s003.docx]

**S3 Table. Means and standard deviations regarding psychophysiological and self-report measures, considering trait-somatic anxiety groups.**

|  |  | LowSG (N=42) | | | | HighSG (N=32) | | | |
| --- | --- | --- | --- | --- | --- | --- | --- | --- | --- |
|  |  | Baseline | | Emotion | | Baseline | | Emotion | |
| Measure | Condition | M | SD | M | SD | M | SD | M | SD |
| LF | Fear | 734.60 | 661.05 | 874.66 | 489.28 | 947.93 | 739.33 | 1014.87 | 625.73 |
|  | Neutral | 681.77 | 441.59 | 1068.01 | 662.84 | 856.04 | 585.61 | 1039.97 | 627.28 |
|  | Happy | 667.16 | 382.75 | 1065.04 | 592.59 | 810.03 | 733.54 | 1087.58 | 637.16 |
| HF | Fear | 557.91 | 641.85 | 535.45 | 496.46 | 435.33 | 385.32 | 429.77 | 326.17 |
|  | Neutral | 525.36 | 382.95 | 526.20 | 409.054 | 407.42 | 345.45 | 414.92 | 371.41 |
|  | Happy | 593.48 | 523.28 | 580.89 | 458.67 | 350.05 | 286.39 | 388.93 | 294.23 |
| LF/HF | Fear | 2.34 | 2.47 | 2.51 | 2.07 | 3.24 | 2.27 | 3.30 | 2.09 |
|  | Neutral | 1.91 | 1.38 | 2.67 | 1.53 | 3.04 | 2.09 | 3.68 | 2.18 |
|  | Happy | 2.20 | 2.27 | 2.86 | 1.92 | 2.80 | 1.61 | 3.51 | 1.81 |
| Happiness | Fear | 49.75 | 24.53 | 24.55 | 26.20 | 47.40 | 20.51 | 29.53 | 23.36 |
|  | Neutral | 49.51 | 24.36 | 41.05 | 26.53 | 49.69 | 20.97 | 51.54 | 24.86 |
|  | Happy | 41.66 | 26.25 | 69.15 | 21.47 | 46.78 | 24.59 | 72.12 | 17.63 |
| Fear | Fear | 5.00 | 13.61 | 40.38 | 32.40 | 7.19 | 13.80 | 34.50 | 33.23 |
|  | Neutral | 6.52 | 15.00 | 0.85 | 2.13 | 9.16 | 16.54 | 4.59 | 11.14 |
|  | Happy | 5.41 | 15.05 | 0.57 | 1.47 | 7.84 | 14.43 | 3.26 | 9.75 |
| Arousal | Fear | 33.21 | 30.88 | 76.97 | 26.03 | 33.49 | 25.38 | 71.38 | 24.28 |
|  | Neutral | 32.81 | 29.20 | 23.99 | 24.26 | 38.09 | 25.20 | 39.03 | 27.65 |
|  | Happy | 28.09 | 25.50 | 67.57 | 24.75 | 43.01 | 24.34 | 69.67 | 21.39 |
