# Supplementary material for: Multidimensional assessment of anxiety through the State-Trait Inventory for Cognitive and Somatic Anxiety (STICSA): From dimensionality to response prediction across emotional contexts
Source: PLoS One. 2022 Jan 25;17(1):e0262960. doi: 10.1371/journal.pone.0262960 (PMC8789173; doi:10.1371/journal.pone.0262960)
Supplement: S3 Table — (DOCX) [file pone.0262960.s004.docx]

**S4 Table. Results of the repeated measures ANOVA, regarding differences between the baselines of the three emotional conditions.**

|  | F | p | ƞ^2^ | Post-hoc tests |
| --- | --- | --- | --- | --- |
| LF | 0.189 | .809 | .003 | NA |
| HF | 0.502 | .606 | .007 | NA |
| LF/HF | 0.910 | .405 | .012 | NA |
| Happiness | 3.352 | .043 | .044 | All n.s. |
| Fear | 0.375 | .688 | .005 | NA |
| Arousal | 0.142 | .845 | .002 | NA |

*Note.* NA: Not applicable; n.s.: nonsignificant.
