# Supplementary material for: Multidimensional assessment of anxiety through the State-Trait Inventory for Cognitive and Somatic Anxiety (STICSA): From dimensionality to response prediction across emotional contexts
Source: PLoS One. 2022 Jan 25;17(1):e0262960. doi: 10.1371/journal.pone.0262960 (PMC8789173; doi:10.1371/journal.pone.0262960)
Supplement: S4 Table — (DOCX) [file pone.0262960.s005.docx]

**S5 Table. Results of the repeated measures ANOVA, regarding differences between groups considering the three emotional conditions at the baseline moment.**

| Variables | | Condition | | | | Group | | | | Condition x Group | | | |
| --- | --- | --- | --- | --- | --- | --- | --- | --- | --- | --- | --- | --- | --- |
|  |  | **F** | **p** | **ƞ^2^** | **Post-hoc tests** | **F** | **p** | **ƞ^2^** | **Post-hoc tests** | **F** | **p** | **ƞ^2^** | **Post-hoc tests** |
| Cognitive Groups | **LF** | 0.223 | .781 | .003 | NA | 0.014 | .905 | .000 | NA | 0.562 | .571 | .008 | NA |
|  | **HF** | 0.464 | .630 | .006 | NA | 0.047 | .829 | .001 | NA | 0.208 | .813 | .003 | NA |
|  | **LF/HF ratio** | 0.962 | .384 | .013 | NA | 0.122 | .727 | .002 | NA | 0.195 | .823 | .003 | NA |
|  | **Happiness** | 3.536 | .037 | .047 | All n.s. | 1.513 | .223 | .021 | NA | 0.419 | .658 | .006 | NA |
|  | **Fear** | 0.531 | .589 | .007 | NA | 2.026 | .159 | .027 | NA | 1.296 | .277 | .018 | NA |
|  | **Arousal** | 0.246 | .758 | .003 | NA | 0.068 | .796 | .001 | NA | 1.922 | .150 | .026 | NA |
| Somatic Groups | **LF** | 2.94 | .745 | .004 | NA | 1.672 | .200 | .023 | NA | 1.219 | .298 | .017 | NA |
|  | **HF** | 0.318 | .728 | .004 | NA | 1.773 | .187 | .024 | NA | 1.339 | .265 | .018 | NA |
|  | **LF/HF ratio** | 0.814 | .445 | .011 | NA | 7.513 | .008 | .094 | HighSG>LowSG | 0.396 | .674 | .005 | NA |
|  | **Happiness** | 2.837 | .068 | .038 | NA | 0.041 | .840 | .001 | NA | 1.252 | .289 | .017 | NA |
|  | **Fear** | 0.376 | .688 | .005 | NA | 0.935 | .337 | .013 | NA | 0.006 | .994 | .000 | NA |
|  | **Arousal** | 0.269 | .742 | .004 | NA | 1.856 | .177 | .025 | NA | 2.407 | .094 | .032 | NA |

*Note.* NA: Not applicable; n.s.: nonsignificant; LowCG: Low trait-cognitive anxiety group; HighCG: High trait-cognitive anxiety group.
