# Supplementary material for: Multidimensional assessment of anxiety through the State-Trait Inventory for Cognitive and Somatic Anxiety (STICSA): From dimensionality to response prediction across emotional contexts
Source: PLoS One. 2022 Jan 25;17(1):e0262960. doi: 10.1371/journal.pone.0262960 (PMC8789173; doi:10.1371/journal.pone.0262960)
Supplement: S5 Table — (DOCX) [file pone.0262960.s006.docx]

**S6 Table. ANOVA's results regarding the psychophysiological measures, considering trait-cognitive anxiety groups.**

|  | | LF | | | | HF | | | | LF/HF Ratio | | | |
| --- | --- | --- | --- | --- | --- | --- | --- | --- | --- | --- | --- | --- | --- |
|  |  | **F** | **p** | **ƞ^2^** | **Simple effects** | **F** | **p** | **ƞ^2^** | **Simple effects** | **F** | **p** | **ƞ^2^** | **Simple effects** |
| Main effects | **Condition** | 0.785 | .458 | .011 | NA | 0.136 | .873 | .002 | NA | 0.177 | .838 | .002 | NA |
|  | **Moment** | 96.052 | p<.001 | .572 | Pre<Post | 6.165 | .015 | .079 | Pre<Post | 72.038 | p<.001 | .500 | Pre<Post |
|  | **Group** | 0.006 | .937 | <.001 | NA | 0.049 | .826 | .001 | NA | 0.105 | .747 | .001 | NA |
| Second-order interaction effects | **Condition x Moment** | 2.850 | .061 | .038 | NA | 1.275 | .281 | .017 | NA | 8.974 | p<.001 | . 111 | Pre: No ≠ across conditions  Post: F<H, p=.049  Pre<Post across conditions, p<.001 |
|  | **Condition x Group** | 1.514 | .224 | .021 | NA | 0.943 | .392 | .013 | NA | .177 | .838 | .002 | NA |
|  | **Moment x Group** | 0.046 | .831 | .001 | NA | 0.007 | .934 | <.001 | NA | .114 | .736 | .002 | NA |
| Third-order interaction effects | **Condition x Moment x Group** | 1.303 | .275 | .018 | NA | 1.730 | .181 | .023 | NA | .101 | .904 | .001 | NA |

*Note.* NA: Not applicable; Pre: evaluation before the emotional induction (baseline); Post: evaluation after the emotional induction (emotion condition); F: Fear condition; H: Happy condition.
