# Supplementary material for: Multidimensional assessment of anxiety through the State-Trait Inventory for Cognitive and Somatic Anxiety (STICSA): From dimensionality to response prediction across emotional contexts
Source: PLoS One. 2022 Jan 25;17(1):e0262960. doi: 10.1371/journal.pone.0262960 (PMC8789173; doi:10.1371/journal.pone.0262960)
Supplement: S6 Table — (DOCX) [file pone.0262960.s007.docx]

**S7 Table. ANOVA's results regarding the self-report measures, considering trait-cognitive anxiety groups.**

|  | | Happiness | | | | Fear | | | | Arousal | | | |
| --- | --- | --- | --- | --- | --- | --- | --- | --- | --- | --- | --- | --- | --- |
|  |  | **F** | **p** | **ƞ^2^** | **Simple effects** | **F** | **p** | **ƞ^2^** | **Simple effects** | **F** | **p** | **ƞ^2^** | **Simple effects** |
| Main effects | **Condition** | 34.978 | p<.001 | .327 | F<N<H | 59.138 | p<.001 | .451 | F>N, F>H | 31.853 | p<.001 | .307 | F>N, F>H |
|  | **Moment** | 0.054 | .818 | .001 | NA | 30.229 | p<.001 | .296 | Pre<Post | 127.321 | p<.001 | .639 | Pre<Post |
|  | **Group** | 0.905 | .345 | .012 | NA | 1.670 | .200 | .023 | NA | 0.072 | .789 | .001 | NA |
| Second-order interaction effects | **Condition x Moment** | 82.991 | p<.001 | .535 | Pre: No ≠ across conditions  Post: F<N<H, p<.001  Pre>Post in F, Pre<Post in H, p<.001 | 60.848 | p<.001 | .458 | Pre: No ≠ across conditions  Post: F>N, F>H, p<.001  Pre<Post in F, Pre>Post in N and H, p<.01 | 54.709 | p<.001 | .432 | Pre: No ≠ across conditions  Post: F>N, H>N, p<.001  Pre<Post in F and H, p<.001 |
|  | **Condition x Group** | 1.292 | .278 | .018 | NA | 1.052 | .352 | .014 | NA | 2.601 | .078 | .035 | NA |
|  | **Moment x Group** | 1.419 | .238 | .019 | NA | 0.147 | .703 | .002 | NA | 1.321 | .254 | .018 | NA |
| Third-order interaction effects | **Condition x Moment x Group** | 0.300 | .742 | .004 | NA | 0.174 | .840 | .002 | NA | 3.414 | .036 | .045 | In N condition: the LowCG had Pre>Post, p=.025; the HighCG had Pre=Post |

*Note.* NA: Not applicable; Pre: evaluation before the emotional induction (baseline); Post: evaluation after the emotional induction (emotion condition); F: Fear condition; H: Happy condition; N: Neutral condition; LowCG: Low trait-cognitive anxiety group; HighCG: High trait-cognitive anxiety group.
