# Supplementary material for: Multidimensional assessment of anxiety through the State-Trait Inventory for Cognitive and Somatic Anxiety (STICSA): From dimensionality to response prediction across emotional contexts
Source: PLoS One. 2022 Jan 25;17(1):e0262960. doi: 10.1371/journal.pone.0262960 (PMC8789173; doi:10.1371/journal.pone.0262960)
Supplement: S7 Table — (DOCX) [file pone.0262960.s008.docx]

**S8 Table. ANOVA's results regarding the psychophysiological measures, considering trait-somatic anxiety groups.**

|  | | LF | | | | HF | | | | LF/HF Ratio | | | |
| --- | --- | --- | --- | --- | --- | --- | --- | --- | --- | --- | --- | --- | --- |
|  |  | **F** | **p** | **ƞ^2^** | **Simple effects** | **F** | **p** | **ƞ^2^** | **Simple effects** | **F** | **p** | **ƞ^2^** | **Simple effects** |
| Main effects | **Condition** | 0.620 | .540 | .009 | NA | 0.062 | .940 | .001 | NA | 0.294 | .746 | .004 | NA |
|  | **Moment** | 96.523 | p<.001 | .573 | Pre<Post | 6.064 | .016 | .078 | Pre<Post | 72.716 | p<.001 | .502 | Pre<Post |
|  | **Group** | 0.770 | .383 | .011 | NA | 2.169 | .145 | .029 | NA | 6.390 | .014 | .082 | LowSG<HighSG |
| Second-order interaction effects | **Condition x Moment** | 3.015 | .052 | .040 | Pre: No ≠ across conditions  Post: F<H, p=.027  Pre<Post across conditions, p<.001 | 1.499 | .228 | .020 | NA | 8.595 | p<.001 | .107 | Pre: No ≠ across conditions  Post: F<H, p=.027  Pre<Post across conditions, p<.01 |
|  | **Condition x Group** | 1.430 | .243 | .019 | NA | 1.454 | .237 | .020 | NA | 0.327 | .721 | .005 | NA |
|  | **Moment x Group** | 5.132 | .026 | .067 | In both groups, Pre<Post, p<.001  No ≠between groups in Pre and Post | 0.001 | .982 | .000 | NA | 6.765 | .011 | .086 | In both groups, Pre<Post, p<.001  In Pre and Post: HighSC > LowSG, p=.05 |
| Third-order interaction effects | **Condition x Moment x Group** | 0.710 | .493 | .010 | NA | 0.441 | .644 | .006 | NA | 0.204 | .816 | .003 | NA |

*Note.* NA: Not applicable; Pre: evaluation before the emotional induction (baseline); Post: evaluation after the emotional induction (emotion condition); F: Fear condition; H: Happy condition; LowSG: Low trait-somatic anxiety group; HighSG: High trait-somatic anxiety group.
