# Supplementary material for: Multidimensional assessment of anxiety through the State-Trait Inventory for Cognitive and Somatic Anxiety (STICSA): From dimensionality to response prediction across emotional contexts
Source: PLoS One. 2022 Jan 25;17(1):e0262960. doi: 10.1371/journal.pone.0262960 (PMC8789173; doi:10.1371/journal.pone.0262960)
Supplement: S8 Table — (DOCX) [file pone.0262960.s009.docx]

**S9 Table. ANOVA's results regarding the self-report measures, considering trait-somatic anxiety groups.**

|  | | Happiness | | | | Fear | | | | Arousal | | | |
| --- | --- | --- | --- | --- | --- | --- | --- | --- | --- | --- | --- | --- | --- |
|  |  | **F** | **p** | **ƞ^2^** | **Simple effects** | **F** | **p** | **ƞ^2^** | **Simple effects** | **F** | **p** | **ƞ^2^** | **Simple effects** |
| Main effects | **Condition** | 36.327 | p<.001 | .335 | F<N<H | 57.825 | p<.001 | .445 | F>N, F>H | 31.831 | p<.001 | .307 | F>N, F>H |
|  | **Moment** | 0.093 | .762 | .001 | NA | 29.467 | p<.001 | .290 | Pre<Post | 125.671 | p<.001 | .636 | Pre<Post |
|  | **Group** | 0.778 | .381 | .011 | NA | 0.303 | .584 | .004 | NA | 1.889 | .174 | .026 | NA |
| Second-order interaction effects | **Condition x Moment** | 82.372 | p<.001 | .534 | Pre: No ≠ across conditions  Post: F<N<H, p<.001  Pre>Post in F, Pre<Post in H, p<.001 | 59.280 | p<.001 | .452 | Pre: No ≠ across conditions  Post: F>N and F>H, p<.001  Pre<Post in F, Pre>Post in N and H, p<.01 | 53.141 | p<.001* | .425 | Pre: No ≠ across conditions  Post: F>N and H>N, p<.001  Pre<Post in F and H, p<.01 |
|  | **Condition x Group** | 0.398 | .672 | .005 | NA | 1.127 | .327 | .015 | NA | 3.053 | 0.50 | .041 | LowSG: F>N, H>N  HighSG: F>N, H>N  LowSG = HighSG in F, N, H |
|  | **Moment x Group** | 2.218 | .141 | .030 | NA | 0.718 | .400 | .010 | NA | 0.514 | .476 | .007 | NA |
| Third-order interaction effects | **Condition x Moment x Group** | 1.485 | .230 | .020 | NA | 0.870 | .421 | .012 | NA | 3.102 | .048 | .041 | Pre: No ≠ across conditions for both groups  Post: F>N and H>N, p<.001 for both groups  Pre ≠ Post in all conditions for LowSG  Pre ≠ Post in all conditions except in the Neutral condition for HighSG  HighSG > LowSG in post-induction of neutral emotion  HighSG > LowSG in pre-induction of happiness |

*Note.* NA: Not applicable; Pre: evaluation before the emotional induction (baseline); Post: evaluation after the emotional induction (emotion condition); F: Fear condition; H: Happy condition; N: Neutral condition.
